# Empowering cancer research in Europe: the EUCAIM cancer imaging infrastructure

**DOI:** 10.1186/s13244-025-01913-x

**Published:** 2025-02-24

**Authors:** Luis Martí-Bonmatí, Ignacio Blanquer, Manolis Tsiknakis, Gianna Tsakou, Ricard Martinez, Salvador Capella-Gutierrez, Sara Zullino, Janos Meszaros, Esther E. Bron, Jose Luis Gelpi, Katrine Riklund, Linda Chaabane, Heinz-Peter Schlemmer, Mario Aznar, Patricia Serrano Candelas, Peter Gordebeke, Monika Hierath, the EUCAIM Consortium, Hanna Leisz, Nicola D’Ascenzo, Miguel Castelo-Branco, Ioanna Chouvarda, Laure Fournier, Sergio Figueiras Gómez, Arcadi Navarro, Nikolaos Papanikolaou, Laurent Barbieri, Jean-Paul Beregi, Georg Langs, David Rodríguez González, Evis Sala, Yiannis Roussakis, Giovanni Di Leo, Antonio López-Rueda, Salvador Pedraza, Javier Blázquez, Carlos Luis Parra Calderón, Silvia Marsoni, Daniel Sáez-Domingo, Marco Aiello, Fredrik Strand, Marie-Christine Jaulent, Joel Hedlund, Laure Saint-Aubert, Carlo Catalano, Regina Beets-Tan, Harald Heese, Angel Alberich-Bayarri, Bram van Ginneken, Marc Van den Bulcke, Daniel Rückert, Emanuele Neri, Philippe Lambin, Gernot Marx, Bengt Persson, Wim Vos, Cátia Sousa Pinto, Patrick Fuhrmann, Maciej Bobowicz, Carles Hernandez-Ferrer, Olivier Humbert, José Miguel Rosell Tejada, Manuela França, Petr Holub, Zdenka Dudova, Isabelle Huys, Matteo Pallocca, Johannes Haybaeck, Patrycja Gazinska, Annabel Seebohm, Tobias Penzkofer, Nils Sandberg, Oscar Gil García, Fernando Martin-Sanchez, Serena Scollen, European Society of Radiology

**Affiliations:** 1 Biomedical Imaging Research Group, Instituto de Investigación Sanitaria La Fe, Valencia, Spain; 2 Universitat Politècnica de València, Valencia, Spain; 3 Foundation for Research and Technology, Hellas, Crete, Greece; 4 Maggioli SPA, Research and Development Lab, Athens, Greece; 5 Universitat de València, Valencia, Spain; 6 Barcelona Supercomputing Center, Barcelona, Spain; 7 EATRIS ERIC, Amsterdam, The Netherlands; 8 Center for IT and IP Law, KU Leuven, Leuven, Belgium; 9 Biomedical Imaging Group Rotterdam, Department of Radiology & Nuclear Medicine, Erasmus MC, University Medical Center Rotterdam, Rotterdam, The Netherlands; 10 Universitat De Barcelona, Barcelona, Spain; 11 Department of Diagnostics and Interventions, Umea Universitet, Umeå, Sweden; 12 EURO-BIOIMAGING ERIC, Med-Hub, Institute of Biostructures and Bioimaging (IBB), Italian National Research Council (CNR), Turin, Italy; 13 Division of Radiology, German Cancer Research Center (DKFZ), Heidelberg, Germany; 14 Matical Innovation, Madrid, Spain; 15 EIBIR Gemeinnutzige Gmbh Zur Forderung Der Erforschung Der Biomedizinischen Bildgebung, Vienna, Austria; 16 Department of Innovation in Medical Physics and Engineering, Instituto Neurologico Mediterraneo (Neuromed), Pozzilli, Italia; 17 Medical Imaging Infrastructure, University of Coimbra, Coimbra, Portugal; 18 Laboratory of Computing, Medical Informatics and Biomedical Imaging Technologies, Aristotle University of Thessaloniki, Thessaloniki, Greece; 19 Department of Radiology, Assistance Publique-Hôpitaux de Paris, Paris, France; 20 BAHIA Software, Santiago De Compostela, Spain; 21 Centre for Genomic Regulation (CRG-CERCA), Barcelona, Spain; 22 Computational Clinical Imaging Group, Champalimaud Foundation, Lisbon, Portugal; 23 Centre National de la Recherche Scientifique, Villeurbanne, France; 24 French College of Radiology Teachers, Paris, France; 25 Department of Biomedical Imaging and Image-Guided Therapy, Medical University of Vienna, Vienna, Austria; 26 Advanced Computation and e-Science, Instituto de Física de Cantabria (IFCA), Consejo Superior de Investigaciones Científicas (CSIC), Santander, Spain; 27 Department of Radiology, Università Cattolica del Sacro Cuore and Fondazione Policlinico Universitario Agostino Gemelli IRCC, Rome, Italy; 28 Department of Medical Physics, German Oncology Center, University Hospital of the European University, Limassol, Cyprus; 29 Radiology Unit, IRCCS Policlinico San Donato, San Donato Milanese, Italy; 30 Fundació Clínic per a la Recerca Biomèdica, Barcelona, Spain; 31 Medical Imaging Department, Hospital Clínic de Barcelona, Barcelona, Spain; 32 Radiology Department, Servicio Madrileño de Salud (SERMAS), Madrid, Spain; 33 Technological Innovation Unit, Virgen del Rocio University Hospital, Servicio Andaluz de Salud (SAS), Seville, Spain; 34 Precision Oncology Unit, Institute for Molecular Oncology, Milano, Italy; 35 Instituto Tecnológico de Informática, Valencia, Spain; 36 Image Processing Laboratory, IRCCS SYNLAB SDN, Naples, Italy; 37 Breast Imaging Unit, Karolinska University Hospital, Stockholm, Sweden; 38 LIMICS, Paris, France; 39 Center for Medical Image Science and Visualization (CMIV), Linköping University, Linköping, Sweden; 40 MEDEXPRIM Labège, Labège, France; 41 Department of Radiological Sciences, University of Rome La Sapienza, Rome, Italy; 42 Department of Radiology, Netherlands Cancer Institute, Amsterdam, The Netherlands; 43 Phillips Research, Hamburg, Germany; 44 QUIBIM, Valencia, Spain; 45 Diagnostic Image Analysis Group, Radboud University Medical Center, Radboud University Medical Center, Nijmegen, The Netherlands; 46 Belgian Cancer Centre, Sciensano, Brussels, Belgium; 47 Institute for AI and Informatics in Medicine, Technical University of Munich, Munich, Germany; 48 Department of Translational Research and New Technologies in Medicine and Surgery at the University of Pisa, Pisa, Italy; 49 Department of the Precision Medicine, Maastricht University, Maastrich, The Netherlands; 50 Clinic for Operative Intensive Medicine and Intermediate Care, University Hospital Aachen (UKA), Aachen, Germany; 51 Department of Cell and Molecular Biology, Uppsala University, Uppsala, Sweden; 52 Radiomics, Flanders, Belgium; 53 International Projects and Affairs Unit, Shared Services of the Ministry of Health (SPMS), Lisbon, Portugal; 54 EGI Foundation, Amsterdam, The Netherlands; 55 Department of Radiology, Medical University of Gdansk (GUMED), Gdansk, Poland; 56 Barcelona Supercomputing Center (BSC), Barcelona, Spain; 57 Department of Nuclear Medicine, French Institute for Research in Computer Science and Automation (INRIA), Nice, France; 58 S2 Grupo, Valencia, Spain; 59 Radiology Department, Centro Hospitalar Universitário de Santo António (CHUP), Porto, Portugal; 60 Biobanking and BioMolecular Resources Research Infrastructure-European Research Infrastructure Consortium (BBMRI-ERIC), Graz, Austria; 61 Institute of Computer Science, Masaryk University, Brno, Czech Republic; 62 Centre for IT and IP Law (CiTiP), KU Leuven, Leuven, Belgium; 63 Istituto Regina Elena - Istituti Fisioterapici Ospitalieri, Rome, Italy; 64 Department of Pathology, Medical University of Innsbruck, Innsbruck, Austria; 65 Biobank Research Group and Section of the Biobank Medical Facility, Łukasiewicz, Polish Center for Technology Development, Wrocław, Poland; 66 Comite Europeen de Coordination des Industries Radiologiques Electromedicales et de Informatique, Brussels, Belgium; 67 Department of Radiology, Charité Berlin University of Medicine, Berlin, Germany; 68 Region Västerbotten, Umeå, Sweden; 69 Technology and Digital Health department, IQVIA Healthcare Spain, Madrid, Spain; 70 National School of Public Health, Carlos III Health Institute of Spain, Madrid, Spain; 71 Human Genomics and Translational Data Department, ELIXIR, ELIXIR Hub, Cambridge, UK

**Keywords:** Cancer research, Imaging, Infrastructure, Artificial intelligence, European Health Data Space

## Abstract

**Abstract:**

Artificial intelligence (AI) is a powerful technology with the potential to disrupt cancer detection, diagnosis and treatment. However, the development of new AI algorithms requires access to large and complex real-world datasets. Although such datasets are constantly being generated, access to them is limited by data fragmentation across numerous repositories and sites, heterogeneity, lack of annotations, and potential privacy issues. The European Cancer Imaging Initiative is a flagship of Europe’s Beating Cancer Plan, aiming to unlock the power of AI for cancer patients, clinicians, and researchers by establishing a federated European infrastructure for cancer images through the EU-funded EUropean Federation for CAncer IMages (EUCAIM) project. This infrastructure, called Cancer Image Europe, builds on the AI for Health Imaging network (AI4HI), established European Research Infrastructures (Euro-BioImaging, BBMRI-ERIC, EATRIS, ECRIN, and ELIXIR), and numerous related partners providing access to research tools, images, and related clinical, pathology and molecular data.

The infrastructure targets clinicians, researchers, and innovators by providing the means to develop and validate data-intensive AI-based and other IT-enabled clinical decision-making systems supporting precision medicine. Common data models, including a linking hyperontology, quality standards, compliance with the FAIR (Findability, Accessibility, Interoperability and Reusability) principles, data annotation, curation and anonymization services are provided to ensure data quality and interoperability, consistency and privacy. In summer 2024, the EUCAIM project released the first prototype of an EU-wide infrastructure, with a comprehensive dashboard integrating applications for dataset discovery, federated search, data access request, metadata harvesting, annotation, secure processing environments and federated processing.

**Critical relevance statement:**

EUCAIM’s federated infrastructure for cancer image data advances medical research and related AI development in Europe. It addresses the current fragmentation and heterogeneity of data repositories is legally compliant, and facilitates collaboration among clinicians, researchers, and innovators.

**Key Points:**

- AI solutions to advance cancer care rely on large, high-quality real-world datasets.
- EUCAIM’s federated infrastructure for cancer image data empowers cancer research in Europe.
- It provides access to research tools, images, and related clinical, pathology and molecular data.

## Introduction

The EUropean Federation for CAncer IMages (EUCAIM) [1] is deploying a pan-European digital federated infrastructure of FAIR anonymized cancer-related images and clinical data from research repositories, clinical trials and other real-world data sources. EUCAIM preserves the sovereignty of data providers while offering an experimentation platform for the development, benchmarking and validation of AI tools, ultimately advancing precision medicine in cancer diagnosis and treatment [2]. EUCAIM addresses the fragmentation and heterogeneity of cancer image repositories leveraging existing initiatives, e.g., the AI4HI initiative [3], European Research Infrastructures and European Commission-funded research projects. The resulting infrastructure platform, Cancer Image Europe, includes a distributed atlas of cancer images and accompanying clinical data covering both common and rare cancer types, along with AI tools and communities of researchers, clinicians and innovators, enabling the development of clinical decision-support systems for precision medicine.

In this paper, we present the principles, design, and first results of the overall pan-European federated infrastructure as an accelerator for medical research and industry innovation [4, 5]. This effort aims to facilitate and foster the development and benchmarking of AI-based cancer management tools towards personalized medicine.

## EUCAIM principles

The infrastructure built by EUCAIM facilitates access to standardized cancer images and related patient data for repositories and healthcare data spaces [6], following these main conditions:

- Alignment: accommodation of existing data and tool repositories with different characteristics and scopes (e.g., different vendors, clinical trials, technical implementations) fulfilling a set of common schemas, standards and quality criteria [7].
- Comprehensive: based on the multidisciplinary involvement of multiple stakeholders (public authorities, hospitals, screening programs, researchers and innovators), and on the inclusion of heterogeneous databases of medical images, as well as on the links to genomics, pathology and molecular data exposing researchers to a high dimensional space.
- Convenience: implementation of a hybrid platform that provides reference nodes to deposit data and federation external nodes to link data along with a central catalogue for the discovery, querying and access to federated datasets, enabling the combination of data sources and their processing in a secure environment.
- Flexible: provision of an adaptable hybrid infrastructure with advanced IT tools and capacities to support an open as-needed basis for emulating clinical trials and AI testing, which implies discovering, assembling and analyzing cancer image datasets.
- Interoperable: utilization of common interoperability mechanisms developed and used in the AI4HI projects (CHAIMELEON, EUCANIMAGE, INCISIVE, PRIMAGE, and ProCANCER-I) [8] and the major Research Infrastructures dealing with cancer data (Euro-BioImaging [9], BBMRI-ERIC [10], EATRIS [11], ECRIN [12] and ELIXIR [13]). The infrastructure will be interoperable with the European Health Data Space (EHDS) [14] components, the initiative of Technical and Experimentation Facilities (TEF) [15], and the developments of the AI Factories under the European High Performance Computing (EuroHPC) Joint Undertaking.
- Secure: implementing a clear data governance model, fully compliant with Ethical Legal and Security considerations, provided by tools and services integrated in a widely adopted Authentication and Authorization Infrastructure, data anonymization, and data traceability with a risk-based approach. The EUCAIM framework also includes privacy-preserving and federated learning services supporting data security and privacy requirements of a Secure Processing Environment [16].
- Extensive: integration of new nodes, data holders and a broad-based spectrum of diseases, covering a broad pan-European geographical distribution, with the development of procedures and tools to facilitate the federation.
- Performance: design of a cutting-edge platform to facilitate rapid observational studies using large-scale data from hospital databases and other existing data sources employing an efficient ETL (Extract, Transform, Load) process to integrate and standardize diverse healthcare datasets.
- Ethical: Access to the data will be subject to the approval of an access committee to ensure compliance with the ethical principles and public good usage purposes.
- Sustainable: setup of a coordinating entity that will host and operate the central services and interact with European and national healthcare systems, public authorities and relevant associations in the business model design, with a long-term vision ensuring data holders’ recognition.

## EUCAIM objectives

The infrastructure addresses a wide range of challenges:

- Setup of a Coordinating Entity hosting and operating the services of the Central Hub while ensuring the application of appropriate legal, recognition, and operative procedures and rules for participation for data holders, service providers and data and service users, enabling the entity to apply for being an active participant in the EHDS.
- Integrate and implement the core services that provide a platform for data discovery, data querying and access to high-quality data stored at the federated nodes.
- Define the common data models, interoperability guidelines, research data management good practices, FAIR principles [17] for data, software and other digital assets, tools and standards for integrating federated data and metadata.
- Conceived from data protection impact assessment, fundamental rights assessment (FRIA) [18] and ethical risk analysis in AI through the Assessment List for Trustworthy Artificial Intelligence (ALTAI) [19] as a platform governed by law and ethics in all its dimensions and throughout its life cycle.
- Integrate key data holders of cancer images, including existing repositories, hospital networks, EU Research Infrastructures and other data holders interested in data sharing.
- Integrate a federated processing environment, including processing tools, federated learning, and computing-intensive frameworks to implement an on-demand GDPR-compliant processing mechanism.
- Monitor the data provisioning, data access, data processing, users, data accesses, and other key metrics related to the repository for reporting, evaluating, and assessing the functionality of the platform.
- Provide governance through the design and implementation of the operating bodies of EUCAIM, which will be in charge of the access, scientific guidance, technical support, training and monitoring of the Cancer Image Europe infrastructure.
- Follow a privacy-by-design approach to integrate with an Authentication and Authorization Infrastructure (AAI) to manage user permissions and implement the required security agreements.
- Set up the Ethical, Legal, and Security framework of Cancer Image Europe, which defines the data access and transfer agreements, the pseudonymization and anonymization procedures, and the legal bounds of the project.
- Define a sustainability plan and implement the necessary structures to operate the repository as a Digital Infrastructure beyond the end of the project, as a long-term European Digital Infrastructure Consortium (EDIC).

## Implementation model

EUCAIM has defined an interoperable framework enabling European data holders (hospitals, biobanks, repositories, and donors) to contribute with FAIR data to build up a metadata catalogue and a distributed repository for annotated images and related data. Such distributed repository is coupled with a federated processing platform for the development and access to AI software. From a centralized dashboard, researchers are able to search and request access to large datasets (images and related clinical-molecular-genetic data) hosted at both the distributed local data holders and the central repository. The platform provides a unique federated imaging research facility for AI testing, experimentation developments and observational studies [20].

Data access will follow ethical, legal, and safety principles (access committee, data protection regulations), followed by signed Data Sharing and Data Transfer Agreements under defined terms of usage with the data holders. All data interactions are traced and registered to guarantee the proper recognition of all the actors involved in the research activities.

**Fig. 1:**
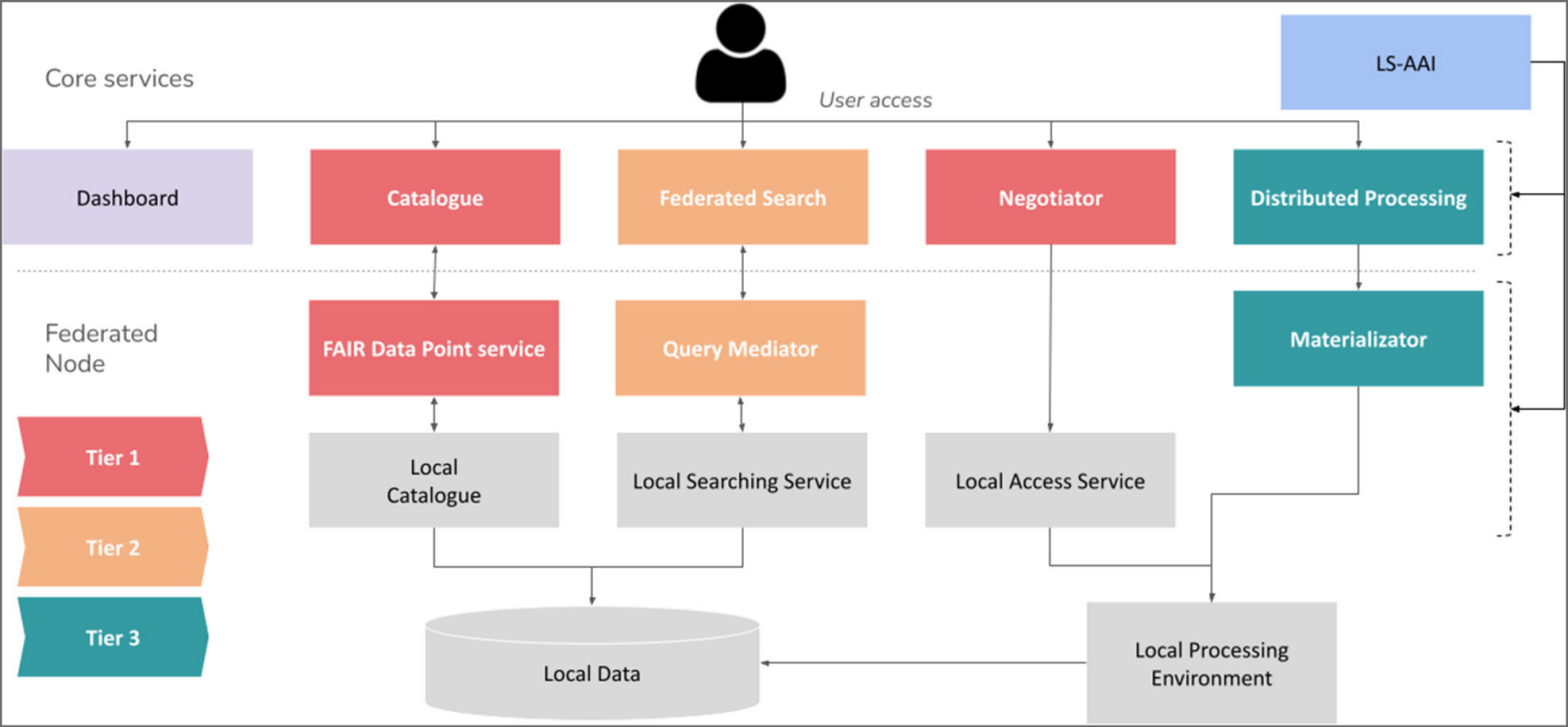
EUCAIM high-level distributed architecture of federated nodes

The hybrid centralized-federated approach, preserving the independence of national, regional, or institutional repositories, providing a centralized governance hub with a cohesive and coherent structure (Fig. 1). Data holders can opt for transferring the data to the EUCAIM secure data repositories, known as Reference nodes, or keeping the data on their premises.

The former will require to sign a Data Transfer Agreement and the latter a Data Sharing Agreement. Federated Data Holders should also sign a Service Level Agreement and provide computing resources. Data Holders connect through the federation layer to the central hub services, which include the Dashboard, the Catalogue, the Federated Search, Access Negotiation and the Distributed Processing.

The centralized components enable data holders who are unable or unwilling to meet the minimum Service Level Agreement for processing and availability to participate in the federation by transferring their data to a designated reference node. The infrastructure is implemented through the following types of services:

- An integration and federation layer comprising services that make the data interoperable and technically link the different local repositories to the core layer.
- A set of centralized resources that allow direct support to individual data collections that are not part of organized and well-structured data-sharing mechanisms.
- A set of core-layer services that provide federated and integrated access to data.

To answer a particular scientific question, users access the platform through the Dashboard, which provides information on data and tools and allows them to access the rest of the applications. Users should sign in to the platform through the Dashboard to obtain valid credentials to access the catalogue, the federated search and the data access request service. The federated search can be used to retrieve the number of subjects that fulfill specific searching criteria based on image modalities, body parts, age, sex or diagnosis (among other standardized terms defined in the EUCAIM data models and the EUCAIM hyperontology) [21]. Access requests are initiated through the catalogue and processed through the negotiator application, which interacts with the Access Committee Access to the data is provided through the platform by means of the federate processing or directly through the data holders.

### Engagement and liaison to EUCAIM

**Fig. 2:**
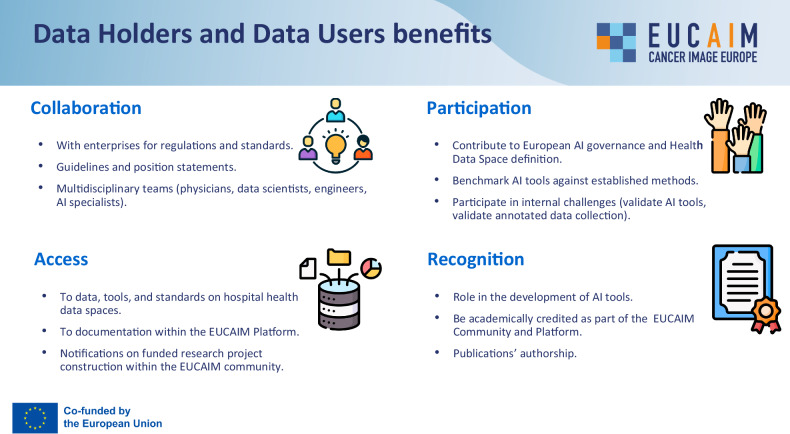
Main benefits of data holders and data users within the EUCAIM infrastructure
- FAIR implementation support. Special attention is paid to ensuring compliance with the FAIR principles [17]:
  - Findability. This is implemented from the data holder side, providing a persistent identifier to the datasets; and from the federated search, providing a way to find the relevant information.
  - Accessibility. Datasets are accessible through a public (but restricted access) endpoint, which will be registered in the EUCAIM catalogue.
  - Interoperability. By being compliant with EUCAIM’s data model and hyperontology, both at the aggregated level of the metadata describing the datasets and the individual data items themselves.
  - Reusability, by providing integration with processing environments and information about the available license/s per dataset.

Data holders’ engagement is a key factor for success. Within the EUCAIM project, an engagement group was established, including the following protocols and activities:

- Engagement and Requirement Analysis: The platform provides a flexible interoperability framework with rules for participation for which differing requirements and expected impacts as well as benefits of the different data holders are considered, based on a model of tiers that describe the federation and compliance with the data models at different levels, enabling a progressive adoption of the EUCAIM procedures.
- Training and evangelization: Data sharing requires motivation, knowledge, and effort. The EUCAIM consortium aims to provide comprehensive information and support, including diverse training material and programs [22], activity networks with representative users, as well as links to other Open Science initiatives. The overall objective is to showcase success stories on the value of sharing, using and reusing data for research. The motivation to contribute to the EUCAIM consortium is strengthened by the provision of trusted frameworks that guarantee data holders their ultimate data control (Fig. 2).

In addition, liaisons with professionals and patient associations are implemented to understand the importance of data sharing and aligning strategies and objectives toward better patient management. Linkage to main EU and international initiatives, including European Strategy Forum on Research Infrastructures (ESFRI)/European Research Infrastructure Consortium (ERIC), the European Health Data Spaces Technical Framework (TEHDAS), the HealthData@EU pilot project, Technical and Experimentation Facilities (TEF), other European projects (e.g., GDI, EOSC4Cancer, UNCAN, IMI Gaia-X, EHDEN, Smart4health, x-eHealth), and the European Digital Innovation Hubs (EDIH) in relevant areas such as AI, Biotechnology, and Pharmaceutics.

### Ethical and legal aspects

A comprehensive and flexible legal model is being implemented to provide a safe, legal ground for data sharing and access [23–25]. The following activities have been performed:

- Construction of the legal operating model. EUCAIM has analyzed the legal constraints of the participating countries and defined the legal ground through Data Transfer and Data Sharing Agreements with the different providers, as well as the Terms of Usage and the data protection and security incident bodies.
- Ethical, anonymization, and GDPR monitoring. EUCAIM has assessed compliance with ethical principles, the terms of usage, ethical considerations of the user, cybersecurity guidelines, and data privacy impact assessments, including the ethics in the application of AI to data and the anonymization principles for the ingestion of data and metadata.
- Data Governance, defining the data access conditions and standards, considering the commitment of both providers and consumers, and the requirements for data traceability.

### Governance and implementation of the central hub

The Central Hub is a virtual organization that consolidates and coordinates access to data, serving three primary roles:

- Define the framework for data sharing from the point of view of data holders and data consumers and coordinate the process.
- Provide a comprehensive central data access portal that harvests the providers’ searchable metadata and manages the access requests.
- Build a central infrastructure to host anonymized data and support AI model development.

The EUCAIM governance is tight to the technical implementation, following these activities:

- Organizational Structure and Procedures. The Central Hub operation required to set committees and a Memorandum of Understanding (MoU) to regulate the operation. The Central Hub provides controlled access for actual access to data. This organizational structure includes elements such as an Access Committee, a Scientific Board, an Ethical and Legal Board, a Steering Board, a Technical Board and other individual positions such as Technical Coordinator, Data Protection Officer, and Scientific Coordinator.
- Rules of Participation and Recognition Models. Participation implies benefits and obligations [26]. EUCAIM has defined a set of rules of participation that check for the minimum contribution, quality standards, type of data, recognition model, certification, and other aspects, required for a member to join.
- Federated Infrastructure core services. The operation of the central hub requires (1) robust and convenient Authentication and Authorization Infrastructure (AAI), considering EduGAIN [27] specifications for cross-border authentication through the Life Sciences AAI [28]; (2) traceability services using Distributed Ledgers in a private Blockchain to support the recognition models and to monitor the fulfillment of the Terms of Usage; (3) a marketplace of processing solutions; and (4) a virtual research environment for image processing and annotation.
- Public Catalogue. The central catalogue will be the entry point to the access portal. EUCAIM has implemented the Central Catalogue with the federated search and Data Access Portal services.

### Service and data interoperability standardization and quality framework

The connection of federated data holders to the registry, federated search and processing services of the central Hub requires defining and implementing procedures, protocols and services. The EUCAIM project defines a minimum viable Federation and Interoperability Framework, and a full model based on three tiers:

- Tier 1: Interoperability is required at the level of the dataset’s aggregated metadata, so the data is findable through the infrastructure’s metadata catalogue.

**Fig. 3:**
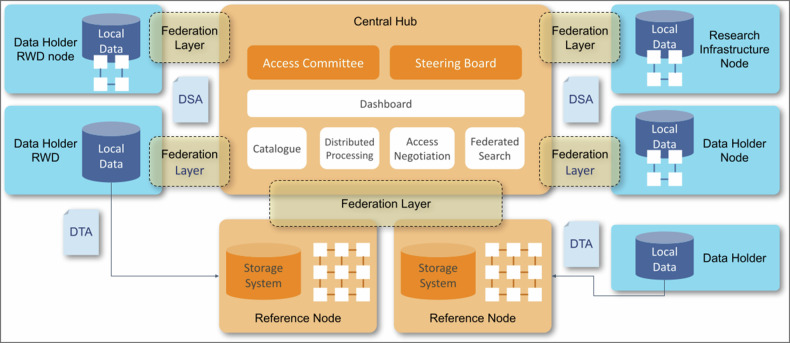
Architecture of the Cancer Image Europe infrastructure
- Tier 2: Interoperability is also implemented at the level of the federated search, ensuring that the metadata is accessible.
- Tier 3: Interoperability also includes integration into the federated processing service.

Figure 3 shows the architecture of the core services, describing more in detail the Federation interoperability layer.

Interoperability requires plugins and tools, which are provided by the Central Hub to the databases for facilitating their federation. The main activities within this strategy are:

- Guidelines for data collection, processing and management.
- Definition of common data and metadata models. EUCAIM has defined a Minimum Common Data Model for the data and metadata, and the protocols, formats, and terminologies for the clinical and imaging data. After the thorough comparison of existing common data models, such as OMOP-CDM, and catalogue specifications (e.g., DCAT-AP for Health and MIABIS), EUCAIM developed a first version of a hyperontology (or hyper-data model) [21] that enables the interoperability of all AI4HI repositories and with any other data collected in the EUCAIM federated repository.
- Guidelines for Data Preprocessing, including (1) tools and criteria for assessing data quality, both at the level of each data item (incomplete, low-resolution, noisy data) and at the dataset level (low number of cases, repeated cases). (2) Data Harmonization Tools for increasing the comparability of different data providers, which employ statistical and IA-driven methods. (3) Data Cleaning tools for detecting and correcting incomplete, incorrect, inaccurate, irrelevant parts of the data and then replacing, modifying, or deleting dirty data. (4) Pseudonymization and/or Anonymization approaches have been defined and agreed upon among the data holders, ensuring compliance with the different regulations. And (5) Data Annotation Tools and Services, already available by consortium members, leveraged and integrated into the EUCAIM infrastructure.
- FAIR principles Definition and Implementation. EUCAIM considers the Research Data Alliance (RDA) recommendations [29] and defines the specific FAIR attributes related to Cancer Imaging data and Health DCAT-AP specification.
- Interoperability plugins for Federated Processing. Two scenarios are considered: If the data provider has already implemented a privacy-preserving data access framework, processing will be directly managed by the provider. If the data provider lacks the necessary resources for the processing, a distributed model based on a sandbox where the user submits the processing remotely will be implemented, and the central hub will act as a broker, facilitating the provision of the required resources for execution.

### Federated data processing and analysis

Federated processing and analysis seamlessly link the processing services of the data holders to the central hub services [30]. The EUCAIM infrastructure focuses on federated learning and federated processing frameworks, as well as on the connection to external e-Infrastructures, by means of:

- Access to High Performance Computing (HPC) and Cloud services across Europe. The federated data processing requires trustable computing resources available next to the data biobanks. In the cases of repositories that do not have direct access to HPC and cloud resources, external providers could be used requiring Service Level Agreements and upfront negotiation.
- Federated orchestration platform. Federated processing will require a framework scheduling, distributing and monitoring workloads by means of middleware on the processing providers to synchronize the workload.
- Federated learning tools. Both synchronous (based on a central parameter server) and asynchronous (fully distributed) federated learning techniques will be supported to run the training in a black-box schema. Currently, EUCAIM has a prototype for synchronous federated learning.
- Enabling trustworthy and privacy-preserving AI and simulation resources. Trustworthiness, implying privacy preservation and control of biases [31], and enhanced privacy-preserving federated training mechanism, ensuring that minimal data is transferred outside of the site boundaries, will be explored in the frame of EUCAIM.

### Use cases for platform expansion and validation

A use case refers to a specific scenario in which the platform is applied to address real-life scientific or clinical questions. These use cases map out the technical, ethical, and legal steps involved, and play a crucial role in defining the requirements for integrating additional datasets, tools, and AI algorithms.

- Data incorporation through open calls to demonstrate the added value of combining existing data from consortium members and newly incorporated data by external parties, which will serve as a basis for federating new cancer images databases.
- Platform usability to promote secondary use of data for research and development activities. These use cases will request access to data and perform a scientifically relevant research study.
- Benchmarking and Quality Control of data, tools and components.
- Validation of the platform through a set of metrics to evaluate the usability, performance, correctness, robustness, and convenience of the platform.

### Business and sustainability models

EUCAIM foresees the participation and use of the infrastructure by researchers and innovators for different settings, including the design and execution of Virtual Clinical Trials, Cancer Screening programs, development and validation of innovative cancer diagnostic tools based on AI (Medical Devices Software (MDSW)), or other experimentations [32, 33].

A comprehensive Sustainability Plan has been defined to maintain and update the computational capabilities, legal agreements, operational framework and related data sharing and access functionalities. During the project implementation, the three sustainability pillars are designed and piloted:

- Business model: identifying and characterizing the needs and motivations of researchers, innovators, patients, public authorities and other relevant stakeholders participating in and using the infrastructure. Different revenue stream models (freemium, pay-per-use, subscription) are designed and will be piloted during the last year of the project.
- Operational model: Devoted to designing the operations workflow to maintain and expand the Central Hub beyond the project’s limit, with all the different departments, positions, equipment, activities, and services.
- Legal Model: Framework to operate as a cross-border infrastructure to fit as an international organization in full compliance with legal and ethical requirements, with a complete and autonomous capacity in any EU Member State.

## Conclusions

The EUCAIM project represents a significant step forward toward empowering medical research across Europe by establishing a federated infrastructure for cancer imaging data. By adhering to FAIR principles and implementing a hybrid centralized-federated model, EUCAIM addresses the challenges of data fragmentation, heterogeneity, and privacy concerns in research. The project’s comprehensive approach, involving multiple stakeholders and integrating various data types, positions it as a powerful platform for advancing precision medicine in cancer diagnosis and treatment. The sustainability and extensibility of the EUCAIM infrastructure are key strengths, with plans to operate as an EDIC beyond the project’s end. By providing a unified platform for data discovery, access, and analysis, EUCAIM facilitates the development and validation of AI-based tools. The project’s commitment to ethical considerations, data protection, and recognition of data holders’ contributions ensures a responsible and collaborative approach to advancing cancer research in Europe.

## Data Availability

The EUCAIM dashboard can be accessed at https://dashboard.eucaim.cancerimage.eu/.

## Abbreviations

AAI: Authentication and authorization infrastructure
AI: Artificial intelligence
AI4HI: AI for Health Imaging network
EDIC: European Digital Infrastructure Consortium
EHDS: European Health Data Space
ERIC: European Research Infrastructure Consortium
EUCAIM: EUropean Federation for CAncer Images
FAIR: Findability, accessibility, interoperability and reusability
HPC: High performance computing
MoU: Memorandum of understanding
TEF: Technical and Experimentation Facilities
